# Anti-Psoriatic Effects of J2H-1802, a Mycophenolate Mofetil and 5-Aminosalicylic Acid Hybrid, in an Imiquimod-Induced Psoriasis-like Mouse Model

**DOI:** 10.3390/pharmaceutics18030380

**Published:** 2026-03-19

**Authors:** Sung-Hoon Park, Ji Hwan Lee, Kyeong-No Yoon, Gabsik Yang, Jason Kim, Ju Young Lee, Kwanghyun Choi, Kiwon Jung, Sumi Lee, Woo-Chan Son, Ki Sung Kang

**Affiliations:** 1 Department of Medical Science, Asan Medical Center, University of Ulsan College of Medicine, Seoul 05505, Republic of Korea; kuroyume27@gmail.com; 2 College of Korean Medicine, Gachon University, Seongnam 13120, Republic of Korea; kleert26@gmail.com (J.H.L.); yanggs@gachon.ac.kr (G.Y.); 3 Department of Dermatology, Seoul National University College of Medicine, Seoul 03080, Republic of Korea; kyeongnoyoon@snu.ac.kr; 4 J2H Biotech Inc., Suwon 16648, Republic of Korea; jsbach@j2hbio.com (J.K.); juyoung105@j2hbio.com (J.Y.L.); ckh@j2hbio.com (K.C.); pharmj@j2hbio.com (K.J.); 5 College of Pharmacy, Seoul National University, Seoul 08826, Republic of Korea; 6 School of Pharmacy and Institute of New Drug Development, Jeonbuk National University, Jeonju 54907, Republic of Korea; chsmath27@jbnu.ac.kr; 7 Department of Pathology, Asan Medical Center, University of Ulsan College of Medicine, Seoul 05505, Republic of Korea

**Keywords:** psoriasis, imiquimod-induced psoriasis model, IL-23/Th17 axis, J2H-1802

## Abstract

**Background/Objectives**: Psoriasis is a chronic immune-mediated inflammatory skin disease characterized by keratinocyte hyperproliferation and systemic inflammatory responses, which are primarily driven by the interleukin (IL)-23/Th17 axis. Although current therapies effectively suppress inflammation, their long-term use is often limited by adverse systemic effects, underscoring the need for safe immunomodulatory agents. This study investigated the anti-psoriatic efficacy of J2H-1802, a novel hybrid compound combining mycophenolate mofetil (MMF) and 5-aminosalicylic acid (5-ASA), in an imiquimod (IMQ)-induced psoriasis-like mouse model. **Methods**: J2H-1802 was orally administered at doses of 125 and 250 mg/kg during IMQ treatment, and its effects were evaluated by conducting clinical assessments, histological analyses, and inflammatory cytokine measurements in the serum and skin tissues. **Results**: J2H-1802 treatment reduced Psoriasis Area and Severity Index (PASI) scores, skin and ear thickness, and splenomegaly in a dose-dependent manner. Histological examination revealed IMQ-induced epidermal hyperplasia attenuation and dermal collagen organization improvement. In addition, J2H-1802 significantly reduced serum tumor necrosis factor-α (TNF-α) levels and suppressed pro-inflammatory cytokine expression, including IL-1β, IL-6, IL-17, and TNF-α, in psoriatic skin. **Conclusions**: J2H-1802 alleviates both local and systemic inflammatory features of psoriasis, suggesting its potential as a therapeutic candidate for targeting IL-23/Th17-mediated inflammatory pathways.

## 1. Introduction

Psoriasis, a chronic autoimmune disease, affects approximately 2% of the global population and is clinically characterized by recurrent, persistent, and well-circumscribed erythrosquamous plaques on the skin [1,2]. Skin lesion characteristics include keratinocyte hyperproliferation; excessive dermal inflammatory infiltration of dendritic cells (DCs), T cells, macrophages, and neutrophils; and increased dermal angiogenesis [3,4]. Although psoriasis pathogenesis is not yet completely understood, it is related to immune tolerance breakdown and excessive inflammatory cytokine production. Interleukin (IL)-23, a cytokine produced by activated dendritic cells, activates undifferentiated T cells and differentiates them into Th17 cells, which are characterized by IL-17 and IL-22 production [5]. These cytokines interfere with keratinocyte transition from the proliferation to the differentiation phase, ultimately resulting in keratinocyte hyperproliferation and epidermal thickening [6,7].

Several treatment options, including conventional systemic agents (e.g., methotrexate, cyclosporine, and retinoids) and biologics targeting key inflammatory pathways, are used to modulate immune responses in psoriasis. Although effective, each therapy requires appropriate safety monitoring based on its specific risk profiles [8,9].

J2H-1802 is a novel hybrid compound synthesized by combining the structural motifs of mycophenolate mofetil (MMF) and 5-aminosalicylic acid (5-ASA). This design aimed to integrate the immunosuppressive and anti-inflammatory activities of MMF with the anti-inflammatory [10,11] and antioxidant properties of 5-ASA [12,13], thereby enhancing its immunoregulatory potential in inflammatory disorders. J2H-1802 is expected to exhibit better pharmacological efficacy than that of its parent compounds through improved stability and synergistic inflammatory pathway modulation. Furthermore, this hybrid strategy was intended to achieve therapeutic efficacy in systemic inflammatory diseases such as psoriasis, while minimizing systemic immunosuppression.

Imiquimod (IMQ), a TLR7/8 receptor ligand, activates macrophages, monocytes, and dendritic cells. When applied to murine skin, IMQ induces clinically notable psoriasis-like skin damage [14,15]. This model is widely used in basic research and its mechanism has been extensively studied [16]. This study evaluated the anti-inflammatory and immunomodulatory effects of J2H-1802, a novel hybrid compound comprising mycophenolate mofetil (MMF) and 5-aminosalicylic acid (5-ASA), in an IMQ-induced psoriasis-like murine model. Specifically, we aimed to determine whether J2H-1802 could improve psoriasis-like skin lesions (erythrosquamous plaques) and epidermal thickening by suppressing IL-23/Th17 axis expression, related inflammatory cytokines, and keratinocyte hyperproliferation.

## 2. Materials and Methods

### 2.1. Chemical

**Scheme 1:**
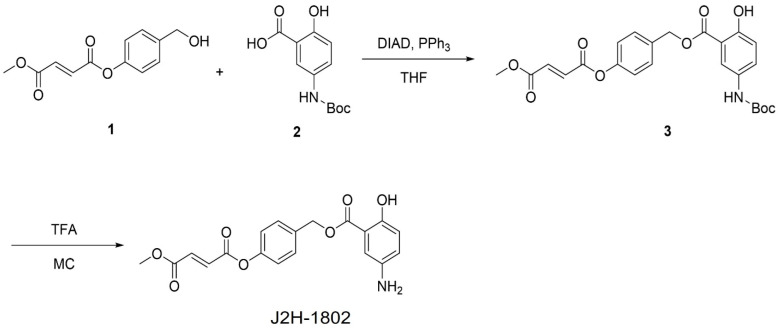
Synthesis of J2H-1802.

J2H-1802 is a synthetic hybrid compound designed to combine the structural elements of monomethyl fumarate (MMF) and 5-aminosalicylic acid (5-ASA) and to combine the potent immunosuppressive and anti-inflammatory activities of MMF with the mucosal-protective and antioxidant effects of 5-ASA. J2H-1802 was synthesized according to the method described in Scheme 1 and was obtained as a white crystalline powder with high purity (>98%), as confirmed by HPLC analysis. The molecular structure was verified by ^1^H-NMR and LCMS. Further details are available in Supplementary Figures S1–S3. For experimental use, J2H-1802 was dissolved in dimethyl sulfoxide (DMSO) to prepare a stock solution (10 mM) and diluted to the indicated concentrations in the culture medium prior to use. All other reagents and solvents were of analytical grade.

### 2.2. Administration

J2H-1802 was provided by J2H Biotech (Suwon, Republic of Korea) and used for the animal experiments described below. Although the prepared samples were stored at 4 °C, they were equilibrated at room temperature before use. J2H-1802 was dissolved in a vehicle containing 10% DMSO and 90% (30% HPbCD) and administered orally once daily for seven days. The doses of 125 mg/kg and 250 mg/kg for efficacy evaluation were determined based on preliminary tolerability assessments and pharmacological criteria.

### 2.3. Animal Experimental Design

Animals were purchased from Daehan Biolink (Chungbuk, Republic of Korea). Female C57BL/6 mice aged 7–8 weeks were obtained and acclimatized for 1 week before the start of the study. All animals were housed under controlled environmental conditions (20–23 °C, approximately 60% relative humidity, 12 h light/dark cycle) with free access to standard rodent diet and water. Mice were randomly divided into four groups (*n* = 6 per group). Group 1 (G1) did not receive any treatment. Group 2 (G2) was treated with 62.5 mg/mouse of Aldara cream (5% imiquimod) to induce psoriasis. Group 3 (G3) received 62.5 mg/mouse of Aldara cream and J2H-1802 at 125 mg/kg, whereas Group 4 (G4) was treated with 62.5 mg/kg/mouse of Aldara cream and J2H-1802 at 250 mg/kg. Detailed information about these compounds is presented in Table 1. To induce psoriasis, the dorsal skin of each mouse was shaved and depilated, and imiquimod treatment was initiated two days later. Aldara cream (62.5 mg/kg/mouse) was applied daily to the dorsal skin and ears at fixed times throughout the experimental period. J2H-1802 administration began one day prior to imiquimod application, and from day 2, topical imiquimod was application in the morning, followed by oral J2H-1802 administration in the afternoon until the end of the study. At the end of the study, the mice were euthanized for sample collection. Blood was collected, and plasma was isolated. Spleen and skin tissues were harvested and stored at −80 °C until analysis. Additionally, skin tissues for histological evaluation were fixed in 10% formalin for 24 h and then stored in a preservation solution at 4 °C until staining. All procedures performed on the animals were approved by the Gachon University Institutional Animal Care and Use Committee (Approval No.: GU1-2023-IA0060-00), and efforts were made to minimize animal numbers and suffering.

### 2.4. Psoriatic Inflammation Evaluation

The severity of psoriasis was evaluated by monitoring the body weight and assessing skin inflammation throughout the study period. Body weight was measured at the same time every other day starting from the beginning of the experiment. To assess cutaneous inflammation, ear thickness and PASI scores were measured. Ear thickness was recorded using a caliper during the experimental period. For PASI scoring, dorsal skin images were taken at baseline and end of the experiment, and the severity was quantified by blinded scoring based on the criteria. Dorsal skin photographs were taken daily throughout the experiment, and PASI scores were determined at the end of the study PASI scoring was performed based on three parameters: erythema, scaling, and thickening, each graded on a 0–4 scale to evaluate the severity of psoriasis [17].

### 2.5. Analysis of Inflammatory Cytokine

Inflammatory cytokines involved with IL-17, IL-1β, IL-6, and TNF-α in tissue and bloods were quantified using ELISA kits according to the manufacturers’ instructions [18]. In addition, the gene expression levels of inflammatory markers were evaluated in the dorsal skin tissues collected at the end of the experiment. Skin tissues were homogenized, and mRNA expression levels were analyzed.

### 2.6. RNA Extraction and Real-Time Polymerase Chain Reaction

Total RNA from the dorsal tissues was extracted using Qiagen RNA mini kit (Qiagen, Hilden, Germany, 74104.) following the manufacturer’s instructions. Equal amounts (1 μg) of total RNA from each sample were converted to complementary DNA (by RNA) to with cDNA RevertAid First Strand cDNA Synthesis Kit (Thermo, Waltham, MA, USA, K1622) in a 20 μL reaction volume. Real-time polymerase chain reaction (PCR) was performed using the AccuPower 2X GreenStar qPCR Mater Mix (Bioneer, Daejeon, Republic of Korea, K6251) from Bioneer (Daejeon, Republic of Korea) according to the manufacturer’s recommendations. The amplification products were analyzed by a melting curve, which confirmed the presence of a single PCR product in all reactions. The primer sequences used in the present study are summarized in Table 2. The amplification program protocol was as follows: 95 °C for 3 min and then 40 cycles consisting of 95 °C for 10 s, 62 °C for 10 s and 72 °C for 10 s. All samples were normalized to glyceraldehyde 3-phosphate dehydrogenase (GAPDH).

### 2.7. Spleen Weight and Size Measurement

At the end of the experiment, the spleen was collected and its weight and size were measured and quantified.

### 2.8. Histological Analysis

The collected skin tissues were stained with H&E and Masson’s trichrome staining. H&E staining was performed to examine skin thickness and morphological changes associated with psoriasis, whereas MT staining was used to evaluate inflammatory changes in the skin tissue [19]. Stained tissues were imaged, and quantitative analysis was conducted using ImageJ software (version 1.53, National Institutes of Health, Bethesda, MD, USA).

### 2.9. Statistical Analysis

Statistical analyses were performed using GraphPad Prism software (version 5.0; GraphPad Software Inc., La Jolla, CA, USA). Quantitative data are presented as the mean ± standard error of the mean (SEM) from at least three independent experiments. Data distribution was evaluated using the D’Agostino–Pearson omnibus normality test. Group differences were analyzed using one-way analysis of variance (ANOVA), followed by Tukey’s post hoc test. Statistical significance was set at *p* < 0.05 significant.

## 3. Results

### 3.1. Body Weight Changes and Psoriasis Severity Index in IMQ-Induced Psoriasis

**Figure 1:**
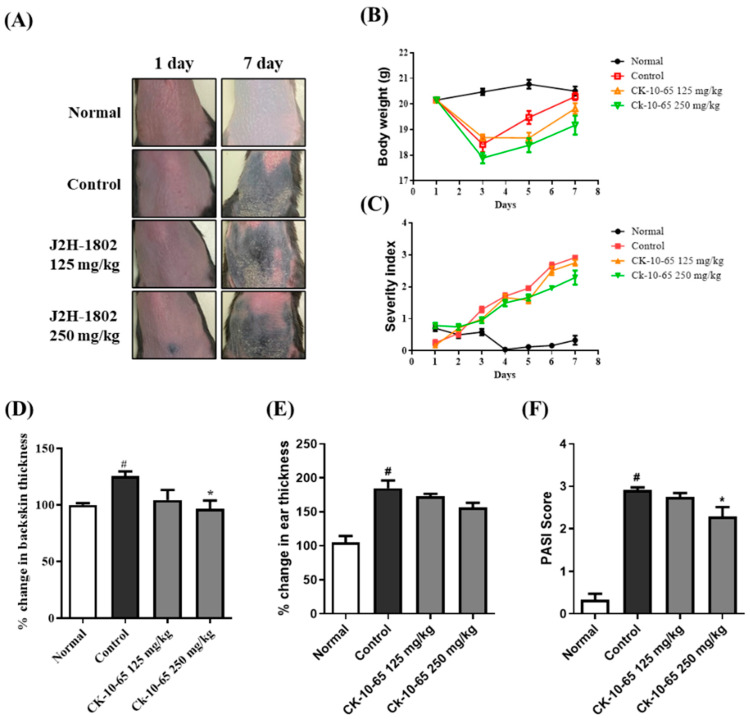
Effect of J2H-1802 on body weight and Psoriasis Area Severity Index (PASI) in imiquimod (IMQ)-induced psoriasis. (**A**) Representative images of IMQ-induced psoriasis on days 1 and 7. (**B**) Weight change observed during the experiment. (**C**) PASI score during experiment, (**D**) back skin thickness at 7 d, (**E**) ear thickness at 7 d, (**F**) PASI score at 7 d. Data are presented as mean ± standard deviation (SD). Statistical analysis was determined by one-way analysis of variance (ANOVA) and Tukey’s multiple comparisons. # *p* < 0.05 vs. normal, * *p* < 0.05 vs. control.

Representative images of dorsal skin lesions are presented in Figure 1A, showing the progression of psoriasis-like skin inflammation in IMQ-treated mice between day 1 and day 7. As shown in Figure 1B, the body weight of mice was measured every alternate during the experimental period. IMQ-treated mice showed weight loss during the first three days after psoriasis induction, followed by gradual recovery. Although the J2H-1802-treated groups exhibited a slight decrease in body weight compared with that of the normal control group, with no statistically significant differences. Furthermore, no acute abnormal behavior was observed throughout the experimental period, further supporting the safety of J2H-1802.

To evaluate psoriasis severity, dorsal skin photographs were taken daily throughout the experiment and Psoriasis Area and Severity Index (PASI) scores were determined at the end of the study. PASI scoring was performed based on three parameters: erythema, scaling, and thickening, each graded on a 0–4 scale to evaluate the severity of psoriasis. As presented in Figure 1C, IMQ application resulted in a time-dependent increase in PASI scores compared with the normal group. As shown in Figure 1D, the dorsal skin thickness in the IMQ control group tended to increase by 1.15-fold compared to that in the normal group. J2H-1802 treatment reduced the dorsal skin thickness by 9.64% and 16.26% at 125 and 250 mg/kg, respectively, in a dose-dependent manner. In addition, ear thickness significantly increased by 1.76-fold in the IMQ control group compared to that in the normal group, whereas J2H-1802 treatment reduced ear thickness by 6.31% and 15.49% at 125 and 250 mg/kg, respectively, in a dose-dependent manner (Figure 1E). On the final day, the PASI score of the IMQ control group was 8.75-fold higher than that of the normal group. J2H-1802 treatment reduced PASI scores by 5.71% at 125 mg/kg and 21.43% at 250 mg/kg in a dose-dependent manner, with statistical significance observed at 250 mg/kg (Figure 1F).

### 3.2. Changes in Spleen Weight and Length

**Figure 2:**
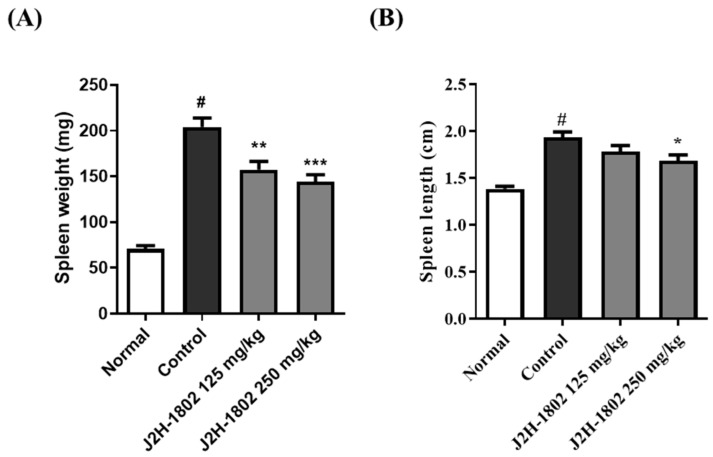
Effect of J2H-1802 on spleen weight and length in imiquimod (IMQ)-induced psoriasis. (**A**) spleen weight at 7 d, (**B**) spleen length at 7 d. Data are presented as mean ± standard deviation (SD). Statistical analysis was determined by one-way analysis of variance (ANOVA) and Tukey’s multiple comparisons. # *p* < 0.05 vs. normal, * *p* < 0.05 vs. control, ** *p* < 0.01 vs. control, *** *p* < 0.001 vs. control.

The spleen is an immune organ that increases in size and weight in response to inflammation. Spleen weight and length were measured at the end of the experimental period. As shown in Figure 2A, the spleen weight of the IMQ control group increased significantly by approximately 2.9-fold compared to that of the normal group. J2H-1802 treatment reduced spleen weight in a dose-dependent manner by 22.76% and 32.02% at 125 mg/kg and 250 mg/kg compared to the IMQ control group, respectively, with both reductions being statistically significant. In addition, as shown in Figure 2B, the spleen length increased significantly in the IMQ control group compared to that in the normal group. J2H-1802 administration decreased the spleen length by 9.18% and 12.24% at 125 and 250 mg/kg, respectively, in a dose-dependent manner, with a statistically significant reduction observed at 250 mg/kg.

### 3.3. Histological Analysis of Skin Tissues

**Figure 3:**
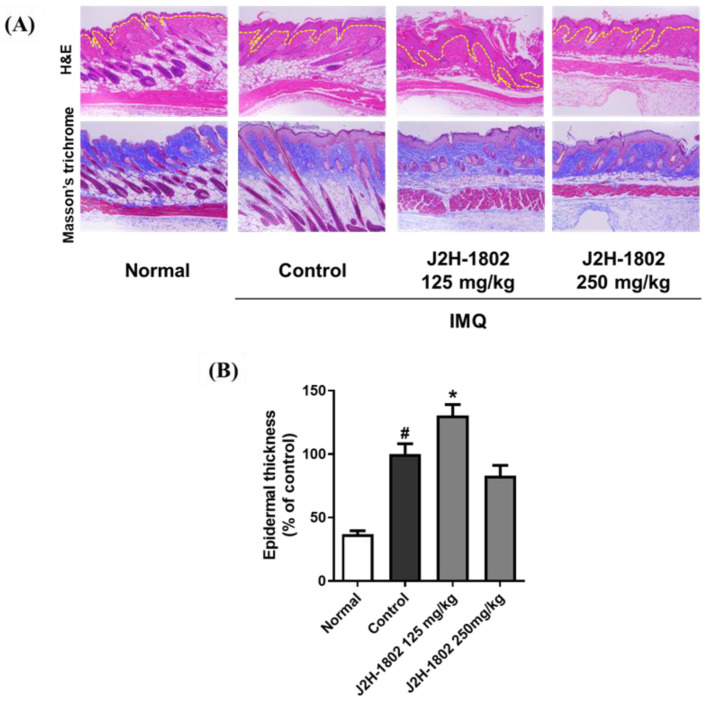
Histological analysis of imiquimod (IMQ)-induced psoriasis skin. (**A**) Hematoxylin & eosin (H&E) and Masson’s trichrome staining images. The yellow line in the H&E staining indicates the dermal border. (**B**) skin epidermal thickness quantitative. Data are presented as mean ± standard deviation (SD). Statistical analysis was determined by one-way analysis of variance (ANOVA) and Tukey’s multiple comparisons. # *p* < 0.05 vs. normal * *p* < 0.05 vs. control.

Histological changes in the dorsal skin were evaluated using hematoxylin and eosin (H&E) and Masson’s trichrome staining. As shown in Figure 3, H&E staining revealed that IMQ exposure markedly stimulated keratinocyte proliferation, resulting in a 2.75-fold increase in epidermal thickness compared to that in the normal group. No notable improvement in epidermal wrinkling or rete ridge formation was observed in the J2H-1802 125 mg/kg group compared to that in the IMQ control group. In contrast, treatment with J2H-1802 at 250 mg/kg alleviated IMQ-induced epidermal thickening and irregular rete ridge morphology.

Masson’s trichrome staining (Figure 3) showed that the normal group exhibited a well-organized collagen fiber structure (stained blue), whereas the IMQ control group showed irregular, loose, and curved collagen fibers, indicating structural disruption. The J2H-1802 125 mg/kg group also displayed irregular collagen alignment, similar to that of the IMQ control. However, J2H-1802 administration at 250 mg/kg improved the structural integrity of collagen fibers, showing a more compact and regular organization compared to that in the IMQ control group.

### 3.4. Cytokine Levels in Blood Analyzed by ELISA

**Figure 4:**
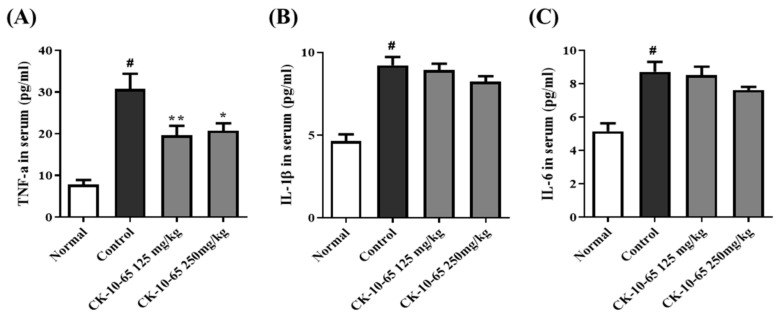
Effect of J2H-1802 on cytokine production in imiquimod (IMQ)-induced psoriasis blood. (**A**) tumor necrosis factor (TNF)-α, (**B**) interleukin (IL)-1β, (**C**) IL-6. Data are presented as mean ± standard deviation (SD). Statistical analysis was determined by one-way analysis of variance (ANOVA) and Tukey’s multiple comparisons. # *p* < 0.05 vs. normal, ** *p* < 0.01 vs. control, * *p* < 0.05 vs. control.

To evaluate the effect of J2H-1802 on cytokine production in IMQ-induced psoriatic mice, cytokine levels of tumor necrosis factor (TNF)-α, interleukin (IL)-1β, and IL-6 were measured using enzyme-linked immunosorbent assay (ELISA). TNF-α levels increased by approximately 4.2-fold in the IMQ control group compared with that in the normal group. J2H-1802 treatment reduced TNF-α levels by 39.56% and 35.99% at 125 mg/kg and 250 mg/kg, respectively. These reductions were significant (Figure 4A). As shown in Figure 4B, IL-1β levels were significantly increased by 1.9-fold in the IMQ control group compared with that in the normal group. J2H-1802 administration reduced IL-1β levels by 3.24% at 125 mg/kg and by 10.85% at 250 mg/kg in a dose-dependent manner, although the reductions were not statistically significant. IL-6 levels were significantly elevated by 1.7-fold in the IMQ control group compared to those in the normal group. J2H-1802 treatment resulted in a dose-dependent decrease of 2.28% and 12.63% at 125 and 250 mg/kg, respectively; however, these changes were not statistically significant (Figure 4C).

### 3.5. Cytokine Levels in Skin Tissues

**Figure 5:**
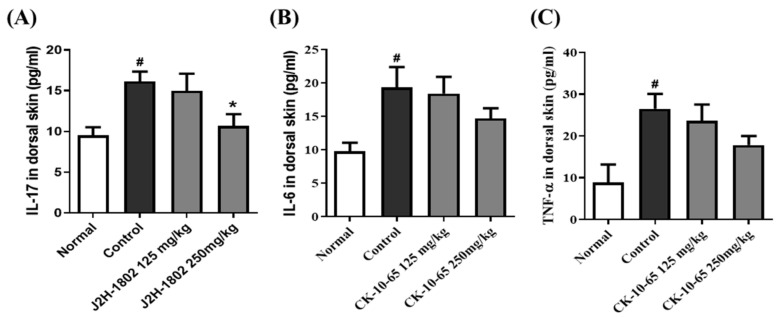
Effect of J2H-1802 on cytokine production in imiquimod (IMQ)-induced psoriasis skin. (**A**) interleukin (IL)-17, (**B**) IL-6, and (**C**) tumor necrosis factor (TNF)-α. Data are presented as mean ± standard deviation (SD). Statistical analysis was determined by one-way analysis of variance (ANOVA) and Tukey’s multiple comparisons. # *p* < 0.05 vs. normal, * *p* < 0.05 vs. control.

To evaluate the effect of J2H-1802 on cytokine production in IMQ-induced psoriatic skin, ELISAs were performed for IL-17, IL-6, and TNF-α. As shown in Figure 5A, IL-17 levels were significantly increased by 1.6-fold in the IMQ control group compared to those in the normal group. J2H-1802 treatment reduced IL-17 levels by 7.1% at the 125 mg/kg dose and by 30.6% at the 250 mg/kg dose, with statistical significance at the 250 mg/kg dose. IL-6 levels in skin tissues tended to increase 2.2-fold in the IMQ control group compared with those in the normal group. J2H-1802 treatment decreased IL-6 levels by 15.5% at 125 mg/kg and 26.12% at 250 mg/kg, showing an overall decreasing trend that was not statistically significant (Figure 5B). In addition, TNF-α levels were elevated by approximately 2.9-fold in the IMQ control group relative to the normal group. J2H-1802 treatment reduced TNF-α levels by 3.32% at 125 mg/kg and by 32.87% at 250 mg/kg. Although reductions were observed across treatment groups, they did not reach statistical significance (Figure 5C).

### 3.6. Cytokine Gene Expression in Skin Tissues

**Figure 6:**
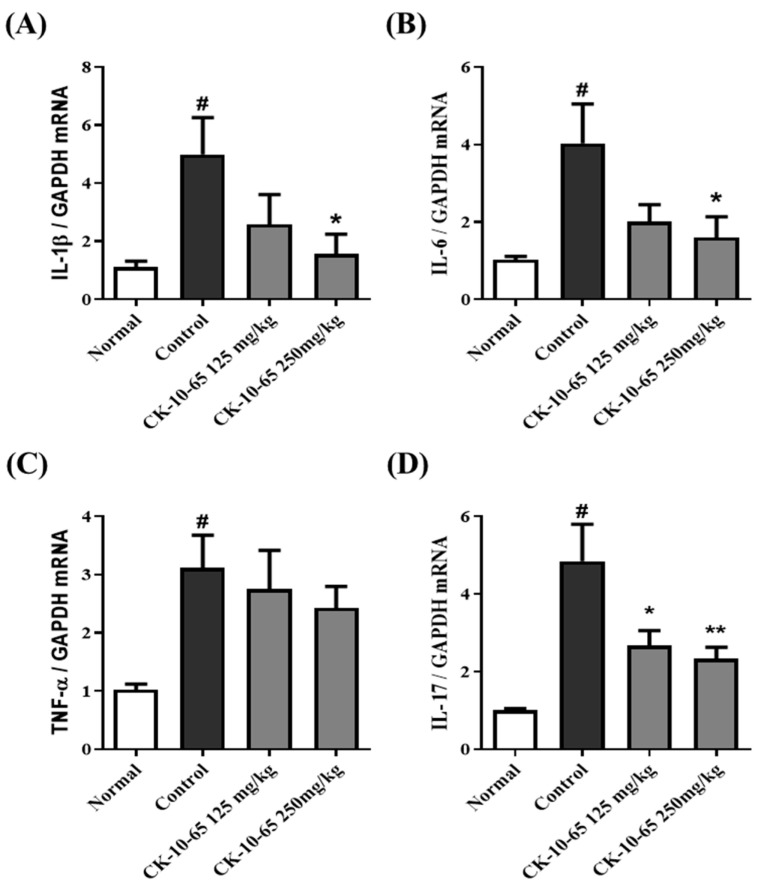
Effect of J2H-1802 on cytokine gene expression in IMQ-induced psoriasis skin. (**A**) IL-1β, (**B**) IL-6, (**C**) TNF-α, (**D**) IL-17. Data are presented as mean ± SD. Statistical analysis was determined by one-way ANOVA and Tukey’s multiple comparisons. # *p* < 0.05 vs. normal, ** *p* < 0.05 vs. control, * *p* < 0.05 vs. control. IL, interleukin; TNF, tumor necrosis factor.

To evaluate the effect of J2H-1802 on cytokine gene expression in IMQ-induced psoriatic skin, mRNA levels of IL-1β, IL-6, IL-17, and TNF-α were measured using real-time PCR. IL-1β expression tended to increase by 3.9-fold in the IMQ control group compared with the normal group. J2H-1802 treatment reduced IL-1β expression by 37.25% at 125 mg/kg and by 75.84% at 250 mg/kg in a dose-dependent manner. Furthermore, the effect reached statistical significance at the 250 mg/kg dose (Figure 6A). IL-6 mRNA levels were elevated by approximately 2.99-fold in the IMQ control group relative to those in the normal group. J2H-1802 treatment reduced IL-6 expression by 28.94% at the 125 mg/kg dose and by a statistically significant 61.89% at the 250 mg/kg dose compared to the control group (Figure 6B). TNF-α gene expression was elevated by approximately 3-fold in the IMQ control group compared with the normal group. J2H-1802 treatment reduced TNF-α expression by 11.73% at 125 mg/kg and by 21.94% at 250 mg/kg in a dose-dependent manner. However, these reductions were not statistically significant (Figure 6C). IL-17 gene expression increased 4.8-fold in the IMQ control group compared to that in the normal group. J2H-1802 treatment reduced IL-17 expression by 47.02% at 125 mg/kg and 54.83% at 250 mg/kg in a dose-dependent manner, and these reductions were statistically significant (Figure 6D).

## 4. Discussion

In this study, we demonstrated that J2H-1802 alleviates clinical severity, local and systemic inflammatory responses, and histological skin damage in an IMQ-induced psoriasis-like dermatitis model. These results indicate that J2H-1802 improves psoriasis pathophysiology through multiple mechanisms, including inflammatory cytokine production modulation and epidermal and dermal structure restoration.

The IMQ-induced psoriasis model is widely used as it activates IL-23/IL-17A, a central pathogenic pathway in human psoriasis, and reproduces key clinical and histopathological features such as erythema, scaling, skin thickening, parakeratosis, and inflammatory cell infiltration [20,21,22,23]. Consistent with previous reports, IMQ treatment in the present study induced psoriatic symptoms, including weight loss, increased PASI score, increased skin thickness on the back and ears, and splenomegaly, during the experimental period. J2H-1802 suppressed the PASI score and skin thickness in a dose-dependent manner, with marked improvement observed in the 250 mg/kg group. The weight loss observed in IMQ-treated mice appears to be related to psoriasis-like model-induced inflammation rather than to the toxicity of the compound itself, a thorough toxicity assessment of the therapeutic’s use is warranted in future studies.

Histopathologically, psoriasis is characterized by hyperkeratosis, parakeratosis, and acanthosis, which result from excessive keratinocyte proliferation driven by interactions with activated immune cells including Th17 cells, plasmacytoid and myeloid dendritic cells, and neutrophils [24,25,26]. Histological analysis confirmed the antipsoriatic effects of J2H-1802. The IMQ-treated group exhibited epidermal thickening, excessive rete ridge elongation, and collagen fiber arrangement disruption, as observed by Masson’s trichrome staining.

Psoriasis is a systemic inflammatory disease. Inflammatory cells and cytokines circulate in various organs and systems and induce a systemic inflammatory response [27]. IL-17, IL-6, IL-1β, and TNF-α induced by skin inflammation spread to other tissues and organs and are associated with systemic inflammatory responses and tissue dysfunction [22,28]. When skin inflammation is activated, inflammatory cells and cytokines spread throughout the body through the bloodstream and promote inflammatory responses in organs and tissues associated with comorbidities such as psoriatic arthritis, depression, obesity, and cardiovascular diseases [29,30].

Splenomegaly is a representative indicator of systemic inflammatory responses observed in the IMQ model [30]. In this study, spleen weight and length significantly increased in the IMQ-treated group. J2H-1802 dose-dependently reduced splenomegaly, supporting the role of J2H-1802 in suppressing not only localized skin inflammation but also systemic immune hyperactivation. Analysis of inflammatory cytokines in systemic and skin tissues showed that IMQ administration tended to increase levels of TNF-α, IL-1β, IL-6, and IL-17, cytokines important in the pathophysiology of psoriasis. J2H-1802 significantly reduced serum TNF-α levels, and levels of IL-1β and IL-6 also showed a consistent decrease. IL-17, IL-6, and TNF-α protein levels in skin tissue also tended to decrease after J2H-1802 treatment, with a significant decrease in IL-17 at the 250 mg/kg dose. Psoriasis is known to play a key role in linking innate and adaptive immunity by regulating the downstream IL-23/Th17 signaling pathway of TNF-α, and STAT3 activation is known to play an important role in Th17 cell differentiation and sustained cytokine production. This suggests that IMQ-activated Th17 and TNF-α related to inflammatory responses were suppressed. Although statistical significance was not achieved for some cytokines, this trend is believed to have biological significance given the high variability of inflammatory cytokines in skin tissue. Furthermore, cytokine-related gene expression analysis showed that J2H-1802 dose-dependently decreased the expression of key inflammatory genes, including IL-1β, IL-6, TNF-α, and IL-17. In particular, the significant decrease in IL-1β, IL-6, and IL-17 mRNA expression at the 250 mg/kg dose suggests that J2H-1802 may modulate the TNF-α axis, which is important in the pathophysiology of psoriasis.

5-Aminosalicylic acid (5-ASA) and mycophenolate mofetil (MMF) have both been studied for the treatment of skin diseases, but their use alone has limitations. 5-ASA exhibits clinical efficacy in psoriasis by modulating keratinocyte differentiation and local inflammatory signaling [31], but its therapeutic effects are primarily limited to skin lesions and are insufficient to address the underlying systemic immune dysfunction in moderate to severe psoriasis [9,32]. In contrast, MMF is known to selectively suppress lymphocyte proliferation by inhibiting inosine monophosphate dehydrogenase (IMPDH) and guanosine nucleotide synthesis. This agent has been used as a systemic immunosuppressant for severe inflammatory and autoimmune skin diseases, including psoriasis [33,34]. However, long-term use is limited by systemic immunosuppression and safety concerns [10]. J2H-1802 was designed to integrate the complementary strengths of the two drugs, overcome their limitations, and provide a comprehensive disease-modifying effect. This dual-mode of action strategy simultaneously modulates local skin inflammation and systemic immune activation, resulting in a balanced and potentially superior therapeutic effect than 5-ASA or MMF alone.

While this study confirmed the anti-psoriatic potential of J2H-1802 in an IMQ-induced psoriasis model, it has three limitations. First, our experimental model is an acute dermatitis model using IMQ, not a chronic disease, which limits direct clinical relevance to human disease. Second, the small sample size in the preclinical efficacy evaluation limits the statistical significance of factors such as cytokines, even despite consistent biological trends. Third, given the compound’s immunomodulatory properties, long-term safety assessment, including hematological and biochemical evaluations, is essential.

In summary, J2H-1802 exhibited anti-psoriatic activity in an IMQ-induced psoriasis model, improved clinical symptoms, reduced splenomegaly, restored the histological skin structure, Statistically validated data showed administration of J2H-1802 significantly reduced PASI scores, ear thickness, spleen weight, and spleen length and gene expression levels of IL-17, IL-1b, and IL-6 in skin tissue at a concentration of 250 mg/kg, and also, significantly reduced serum and gene of TNF-α levels in animals. These results suggest that J2H-1802 is potential anti-psoriasis to improve psoriasis pathophysiology through modulation of inflammation centered on the IL-23/Th17 axis and TNF-α signaling. This study focused on evaluating the anti-psoriatic efficacy of J2H-1802. Future studies will focus on elucidating the mechanism of action of J2H-1802, including its regulation of inflammation-related STAT3 phosphorylation, NF-κB activation, and Th17 cell differentiation. Furthermore, comparative studies with MMF and 5-ASA will assess the pharmacological benefits and long-term safety of the hybrid formulation, and evaluation in a chronic psoriasis model will advance its clinical application.

## Figures and Tables

**Table 1:** Animal group and administration information.

Group	Sample	Dose	Administration	Dosing Frequency	*n*
G1	-	Saline	PO	QD × 7 d	6
G2	-	Saline	PO	QD × 7 d	6
G3	J2H-1802	125 mg/kg (10 mL/kg)	PO	QD × 7 d	6
G4	J2H-1802	250 mg/kg (10 mL/kg)	PO	QD × 7 d	6

QD, quaque die; PO, per oral.

**Table 2:** Sequence of primer used for real-time qPCR.

Gene	Sequence	
IL-1β	Forward	CGG ACT CCG CCA AGT CTA AG
	Reverse	CCA CAG GTA TTT TGT CG
IL-6	Forward	GAG GAT ACC ACT CCC AAC AGA CC
	Reverse	AAG TGC ATC ATC GTT GTT CAT ACA
TNF-α	Forward	CGT CAG CCG ATT TGC TAT CT
	Reverse	CGG ACT CCG CCA AGT CTA AG
IL-17	Forward	ATC CCT CAA AGC TCA GCG TGT C
	Reverse	GGG TCT TCA TTG CGG TGG TGG AGA G
GAPDH	Forward	GAA GGT CGG TGT GAA CGG AT
	Reverse	CCC ATT TGA TGT TAG CGG GAT

## Data Availability

All data generated or analyzed during this study are included in this published article. Further information may be obtained from the corresponding author upon reasonable request.

## Supplementary Materials

The following supporting information can be downloaded at: https://www.mdpi.com/article/10.3390/pharmaceutics18030380/s1, Figure S1. ^1^H NMR spectrum of J2H-1802 (400 MHz, solvent: DMSO-d6). Figure S2. Mass spectrum of J2H-1802 (ES+). Figure S3. HPLC chart of J2H-1802.

## Abbreviations

The following abbreviations are used in this manuscript:

IMQ	Imiquimod
PASI	Psoriasis Area and Severity Index
TNF-α	Tumor necrosis factor-α
IL-1β	Interleukin-1β
IL-6	Interleukin-6
Il-17	Interleukin-17
