# Supplementary material for: Anti-Psoriatic Effects of J2H-1802, a Mycophenolate Mofetil and 5-Aminosalicylic Acid Hybrid, in an Imiquimod-Induced Psoriasis-like Mouse Model
Source: Pharmaceutics. 2026 Mar 19;18(3):380. doi: 10.3390/pharmaceutics18030380 (PMC13029267; doi:10.3390/pharmaceutics18030380)
Supplement: Supplementary file 1 [file pharmaceutics-18-00380-s001.zip › pharmaceutics-4152131-supplementary.pdf]

pharmaceutics

Supplementary Materials: Anti-Psoriatic Effects of J2H-1802, a Mycophenolate Mofetil and 5-Aminosalicylic Acid Hybrid, in an Imiquimod-Induced Psoriasis-like Mouse Model

Sung-Hoon Park, Ji Hwan Lee, Kyeong-No Yoon, Gabsik Yang, Jason Kim, Ju Young Lee, Kwanghyun Choi, Kiwon Jung, Sumi Lee, Woo-Chan Son and Ki Sung Kang

Synthesis of J2H-1802.

1. Synthesis of 4-(((5-((tert-butoxycarbonyl)amino)-2-hydroxybenzoyl)oxy)methyl)phenyl methyl fumarate (3)

To a solution of 4-(hydroxymethyl)phenyl methyl fumarate (1) (5 g, 21.2 mmol), 5-((tert-butoxycarbonyl)amino)-2-hydroxybenzoic acid (2) (6.43 g, 25.4 mmol) and triphenylphosphine (6.66 g, 25.4 mmol) in THF (100 mL), Diisopropyl azodicarboxylate (5 mL, 25.4 mmol) was slowly added under ice bath. The ice bath was removed, and the mixture was stirred at room temperature for 16 hours. The reaction mixture was concentrated under reduced pressure, after that 50 mL of isopropyl alcohol was added and stirred. The resulting solid was filtered and washed with 30 mL of isopropyl alcohol and 50 mL of heptane. And then vacuum dried at room temperature for 16 hours, 8.2 g (82%) of 4-(((5-((tert-butoxycarbonyl)amino)-2-hydroxybenzoyl)oxy)methyl)phenyl methyl fumarate was obtained.

2. Synthesis of 4-(((5-amino-2-hydroxybenzoyl)oxy)methyl)phenyl methyl fumarate (J2H-1802)

To a solution of 4-(((5-((tert-butoxycarbonyl)amino)-2-hydroxybenzoyl)oxy)methyl)phenyl methyl fumarate (3) (8 g, 17.0 mmol) in dichloromethane (40 mL), trifluoroacetic acid (15.1 mL, 204 mmol) was added at room temperature and the mixture was stirred for 3 hours. The reaction mixture was basified with sat. $NaHCO_3$ solution and extracted with dichloromethane. The organic layer was dried with $MgSO_4$, filtered, and concentrated. 100 mL of heptane was added, stirred for 30 minutes, and filtered. And then vacuum dried at 50°C for 4 hours, 5.9 g (94%) of 4-(((5-amino-2-hydroxybenzoyl)oxy)methyl)phenyl methyl fumarate was obtained.

^{1}H NMR (400 MHz, DMSO) δ 7.56 (d, J = 8.4 Hz, 2H), 7.28 (d, J = 8.8 Hz, 2H), 7.03 (d, J = 3.2 Hz, 1H), 6.83 (dd, J = 8.8 Hz, 2.8 Hz, 1H), 6.72 (d, J = 8.8 Hz, 1H), 5.37 (s, 2H), 4.82 (s, 2H), 3.79 (s, 3H); HRMS (ES+) m/z found 372.1090 [M+H] (calcd for C19H17NO7 371.1005) ; HPLC purity, 99.5%.

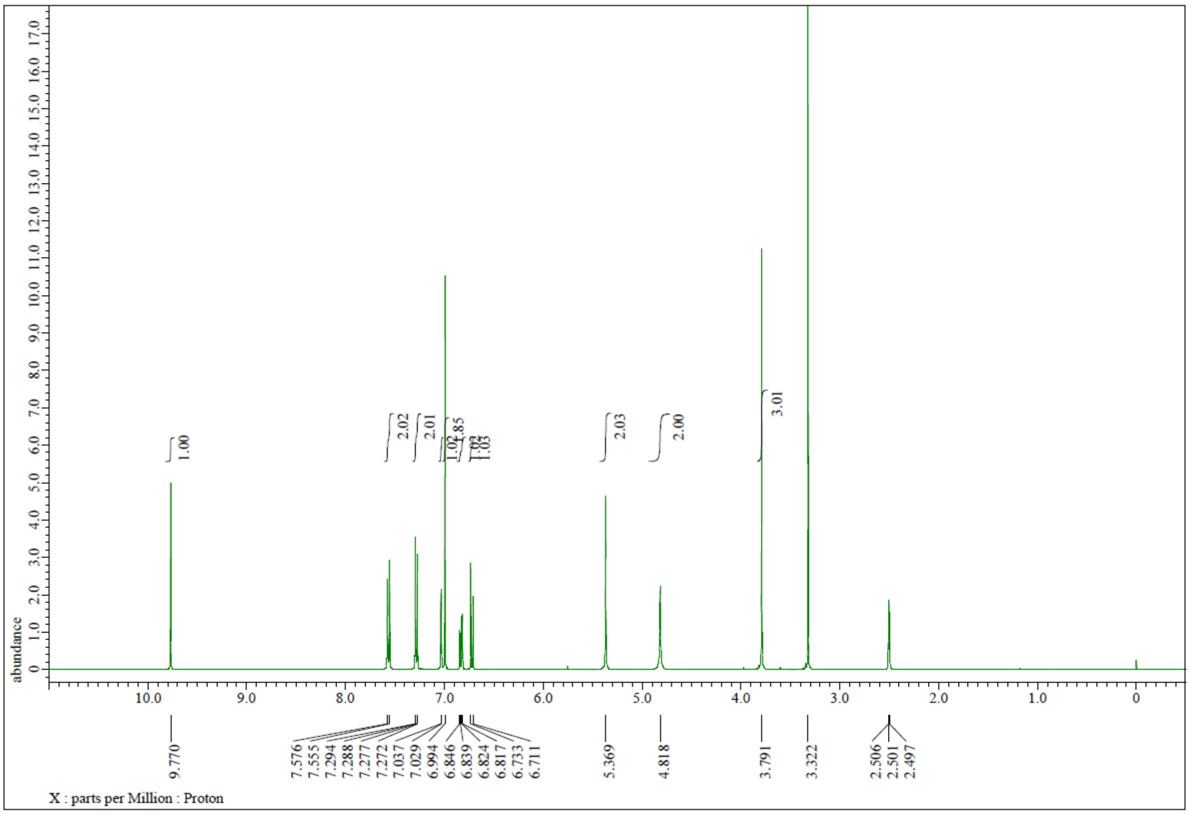

Figure S1. **¹H NMR spectrum of J2H-1802 (400 MHz, solvent: DMSO-*d6*)**

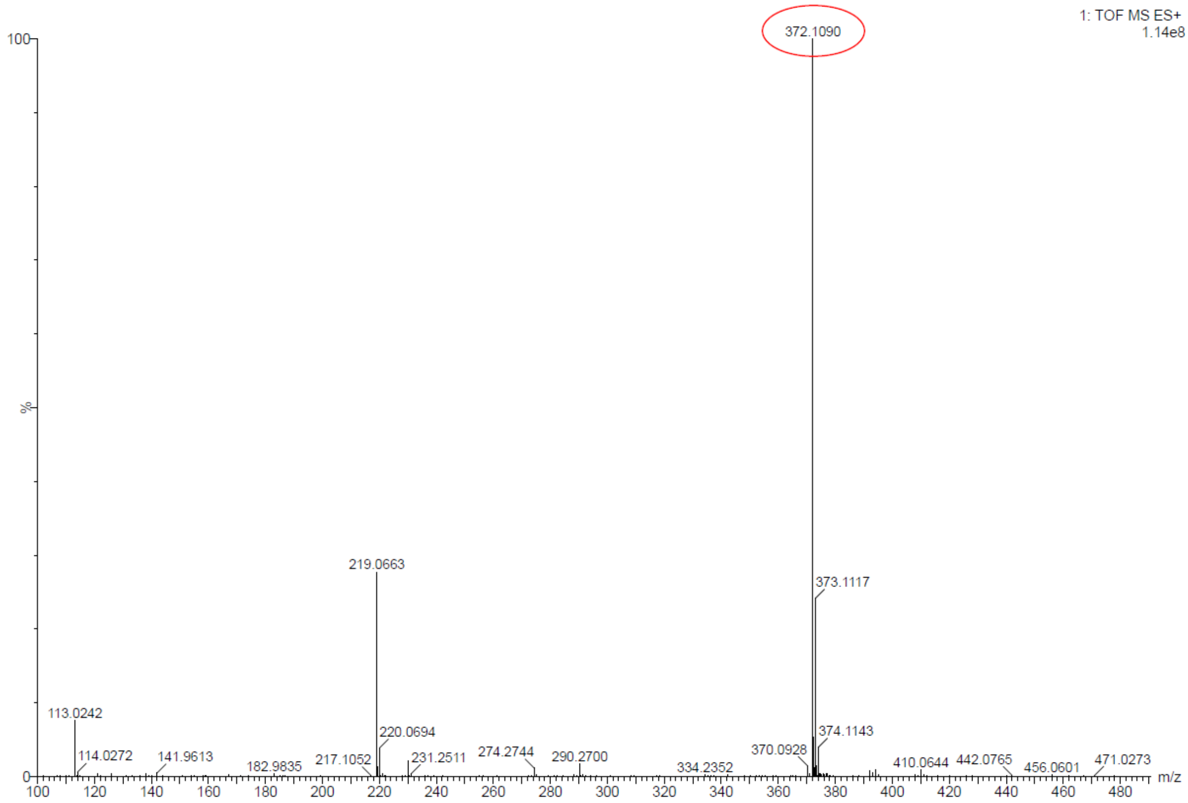

Figure S2. Mass spectrum of J2H-1802 (ES+)

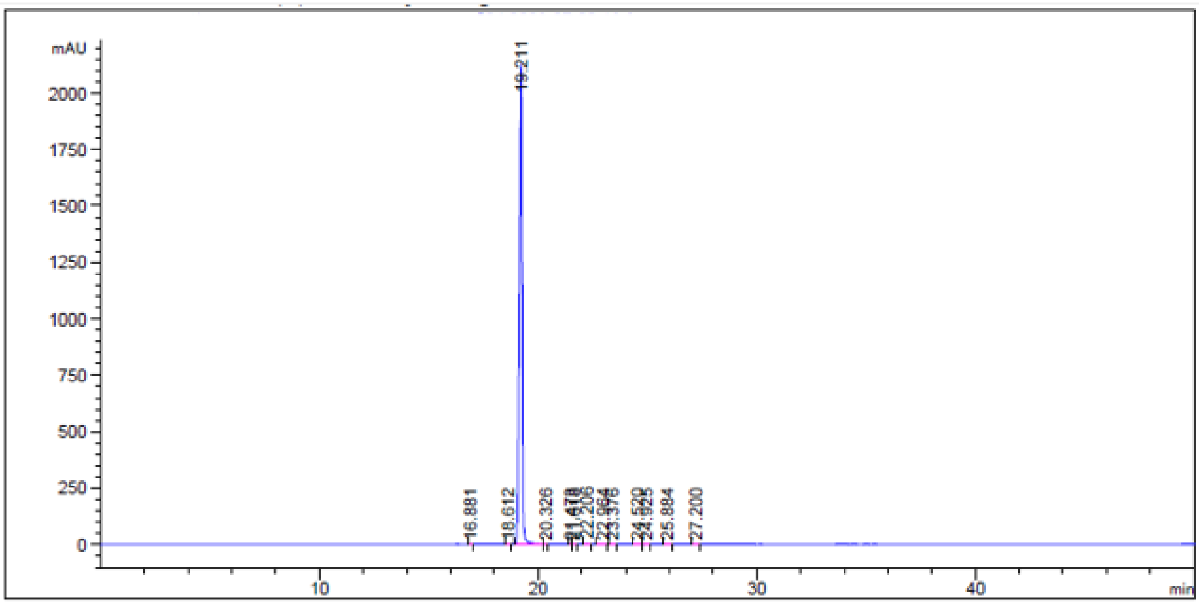


```
=====================================================================
                        Area Percent Report
=====================================================================

Sorted By             :      Signal
Multiplier            :      1.0000
Dilution              :      1.0000
Do not use Multiplier & Dilution Factor with ISTDs


Signal 1: VWD1 A, Wavelength=235 nm
```

Peak #	RetTime [min]	Type	Width [min]	Area [mAU*s]	Height [mAU]	Area %
1	16.881	BB	0.0698	10.12508	2.19697	0.0572
2	18.612	BBA	0.1027	1.84274	2.73135e-1	0.0104
3	19.211	BBA	0.1293	1.76128e4	2112.46924	99.5356
4	20.326	BB	0.1201	2.14385	2.83842e-1	0.0121
5	21.473	BBA	0.0882	2.20046	3.99993e-1	0.0124
6	21.618	BBA	0.0836	2.26204	4.43121e-1	0.0128
7	22.206	BBA	0.1214	19.22101	2.40031	0.1086
8	22.964	BBA	0.1119	14.64117	2.03505	0.0827
9	23.376	BB	0.1089	2.27503	3.27969e-1	0.0129
10	24.520	BB	0.1165	10.62424	1.46627	0.0600
11	24.925	BB	0.1171	2.78022	3.64086e-1	0.0157
12	25.884	BB	0.1206	2.72773	3.43750e-1	0.0154
13	27.200	BBA	0.1117	11.33354	1.65942	0.0640

Figure S3. HPLC chart of J2H-1802
